# Translation, Cross-Cultural Adaptation, and Validation of the Measure of Intermittent and Constant Osteoarthritis Pain (ICOAP) Measure into Brazilian Portuguese for Individuals with Knee Osteoarthritis

**DOI:** 10.3390/healthcare13111283

**Published:** 2025-05-29

**Authors:** Gabriela Nascimento de Santana, Patrícia Gabrielle dos Santos, Luciano Bernardes Leite, Pedro Forte, José Eduardo Teixeira, Christiano Eduardo Veneroso, Almir Vieira Dibai-Filho, Cid André Fidelis-de-Paula-Gomes

**Affiliations:** 1 Postgraduate Program in Rehabilitation Sciences, Nove de Julho University, São Paulo 04014-000, SP, Brazil; gabynascimento45@hotmail.com (G.N.d.S.); patgabsantos@gmail.com (P.G.d.S.); cid.andre@gmail.com (C.A.F.-d.-P.-G.); 2 Department of Physical Education, Federal University of Viçosa—Viçosa Campus, Viçosa 36570-900, MG, Brazil; luciano.leite@ufv.br; 3 Department of Sports, Higher Institute of Educational Sciences of the Douro, 4560-708 Penafiel, Portugal; pedromiguelforte@gmail.com; 4 CI-ISCE, ISCE Douro, 4560-708 Penafiel, Portugal; 5 Department of Sports, Instituto Politécnico de Bragança, 5301-854 Bragança, Portugal; 6 Research Center for Active Living and Wellbeing (LiveWell), Instituto Politécnico de Bragança, 5301-854 Bragança, Portugal; 7 Department of Sports Sciences, Polytechnic of Guarda, 6300-559 Guarda, Portugal; 8 Department of Sports Sciences, Polytechnic of Cávado and Ave, 4750-810 Guimarães, Portugal; 9 SPRINT—Sport Physical Activity and Health Research & Inovation Center, 6300-559 Guarda, Portugal; 10 Postgraduate Program in Physical Education, Universidade Federal do Maranhão, São Luís 65080-805, MA, Brazil; christiano.veneroso@ufma.br (C.E.V.); almir.dibai@ufma.br (A.V.D.-F.)

**Keywords:** knee osteoarthritis, questionnaires, chronic pain

## Abstract

**Background:** A comprehensive understanding of chronic pain is crucial for assessing its impact on knee osteoarthritis (KOA). This study aimed to translate, cross-culturally adapt, and validate the ICOAP into Brazilian Portuguese for individuals with KOA. **Methods:** A total of 133 individuals with KOA participated. Phase 1 involved the translation and cross-cultural adaptation of the ICOAP into Brazilian Portuguese. In Phase 2, the pre-final version was tested, and in Phase 3, the final version was validated with 103 participants. Convergent validity was assessed using Spearman’s correlation with the Numeric Rating Pain Scale (NRPS), the International Knee Documentation Committee (IKDC), and the Short Form 36 Health Survey (SF-36). Internal structure was evaluated through exploratory and confirmatory factor analyses using fit indices: chi-square/degrees of freedom (χ^2^/df), root mean square error of approximation (RMSEA), standardized root mean square residual (SRMR), comparative fit index (CFI), and Tucker–Lewis index (TLI). Internal consistency was assessed with Cronbach’s alpha (α), and floor and ceiling effects were examined. A subsample of 53 participants was used to assess test–retest reliability through the intraclass correlation coefficient (ICC), standard error of measurement (SEM), and minimum detectable change (MDC). **Results:** The ICOAP showed adequate fit indices. Reliability analysis demonstrated satisfactory internal consistency and test–retest reliability. However, only the constant pain domain exhibited convergent validity. **Conclusions:** The Brazilian Portuguese version of the ICOAP consists of two independent domains with good internal consistency and reliability. However, only the constant pain domain showed appropriate convergent validity.

## 1. Introduction

Although it varies among individuals and different stages of knee osteoarthritis (KOA), pain remains the most prominent symptom and one of the primary drivers of clinical decision-making [1,2]. The neurobiological mechanisms underlying osteoarthritis-related pain are complex, involving both peripheral processes and central nervous system pathways [1,2]. These mechanisms are influenced by individual contextual factors and various psychosocial variables [1,2]. This multifactorial complexity makes assessing osteoarthritis-related pain a challenging task, often regarded as highly precarious in clinical practice [1].

One of the most comprehensive patient-reported outcome measures (PROMs) for osteoarthritis is the Intermittent and Constant Osteoarthritis Pain (ICOAP) scale [3]. Developed through focus groups, it assesses the severity of pain and its impact on the quality of life of individuals with knee and hip osteoarthritis [3,4]. Notably, it is the only scale specifically designed to distinguish between constant and intermittent pain in this population. The ICOAP can also be used to monitor disease progression, treatment response, and the potential need for joint replacement [2,4]. Due to its high reliability in assessing patients’ conditions, the ICOAP has been translated into multiple languages and has been widely adopted in studies involving diverse populations [1,3,4].

None of the pain assessment tools currently available for the Brazilian population provide the specific analytical capabilities offered by the ICOAP questionnaire. This instrument gives clinicians and researchers a novel assessment tool with strong psychometric support, specifically designed to comprehensively evaluate both intermittent and constant pain, along with its impacts on mood, sleep, quality of life, and functionality in individuals with KOA. Therefore, this study aims to translate, cross-culturally adapt, and evaluate the psychometric properties of the ICOAP for individuals with knee osteoarthritis (KOA). 

## 2. Materials and Methods

### 2.1. Study Design and Ethical Considerations

This methodological study involved the translation and cross-cultural adaptation of the ICOAP-Knee. It received approval from the Ethics Committee of Nove de Julho University, São Paulo, under approval number 32671720.7.3001.5087 on 20 June 2020. Authorization to translate and adapt the questionnaire was obtained from the original authors via email. The study adhered to established guidelines for the cross-cultural adaptation of self-reporting measures and complied with the Consensus-Based Standards for the Selection of Health Measurement Instruments (COSMIN) [5,6].

The study was conducted in accordance with the Declaration of Helsinki. All participants received both oral and written information about the study before providing informed consent, which was obtained in verbal and written forms. The study was conducted between January 2020 and October 2023 and comprised three phases: Phase 1: Translation and cross-cultural adaptation of the ICOAP-Knee into Brazilian Portuguese; Phase 2: Pretesting of the pre-final Brazilian Portuguese version of the ICOAP-Knee; Phase 3: Validation of the final cross-culturally adapted Brazilian Portuguese version of the ICOAP-Knee.

### 2.2. Intermittent and Constant Osteoarthritis Pain (ICOAP)

The ICOAP consists of 11 questions divided into two domains. One domain includes five questions about constant pain, while the other comprises six questions focused on intermittent pain, defined as “pain that comes and goes”.

For the constant pain domain, 0 = nothing/no constant pain; 1 = mildly, 2 = moderately, 3 = severely, and 4 = extremely. For the intermittent pain domain, exclusively for question 7, 0 = never/no pain that comes and goes, 1 = rarely, 2 = sometimes, 3 = often, and 4 = very often. For the others, 0 = nothing/no pain that comes and goes, 1 = slightly, 2 = moderately, 3 = severely, and 4 = Extremely [2,7].

All questions are scored using a Likert scale ranging from 0 to 4 points. A higher score indicates a greater presence of constant and/or intermittent pain. The final absolute score is converted into a scale from 0 to 100 for both domains, using the formulas (Total constant pain score/20) × 100 and (Total pain score/24) × 100 [2,7].

### 2.3. Participants

A total of 133 individuals diagnosed with KOA participated in all phases of the study. Of these, 30 individuals took part in Phase 2, which involved testing the pre-final Brazilian Portuguese version of the ICOAP-Knee. The remaining 103 individuals participated in Phase 3, which focused on validating the final cross-culturally adapted version of the ICOAP-Knee.

The sample size was determined based on COSMIN recommendations, which suggest a minimum of seven participants per item of the instrument, provided that the total sample includes at least 100 individuals [6].

Participants were required to meet the following inclusion criteria: any gender, aged over 40 years, with a medical diagnosis of KOA, a complaint of pain and/or functional impairment in the knee lasting at least 12 weeks, morning stiffness, and a pain intensity score greater than three on the Numeric Rating Scale [2,3]. Additionally, participants had to be fluent in Brazilian Portuguese and literate, with the ability to read and write in the language. Individuals scoring below the cutoff points on the Mini-Mental State Examination—23 or lower for those with higher education and 17 or lower for those with lower education—were excluded [8]. Participants were also excluded if they had undergone knee surgery, received intra-articular corticosteroid or hyaluronic acid injections during the study period, or were using adrenocortical hormones or non-steroidal anti-inflammatory drugs (NSAIDs) for other medical conditions such as cancer or diabetes [2,3].

### 2.4. Phases 1 and 2

Phase 1, which involved the translation and cross-cultural adaptation of the ICOAP-Knee into Brazilian Portuguese, and Phase 2, which consisted of testing the pre-final version, were conducted following the guidelines proposed by Beaton et al. [5]. These phases were executed in five distinct stages.

Stage 1: Two independent translators translated the original English version of the ICOAP-Knee into Brazilian Portuguese. The first translator was a physiotherapist with ten years of clinical experience in the field, while the second was an English language teacher with twenty years of experience in translation. Both translators were fluent in Portuguese and English.

Stage 2: After the independent translation process, the two translators, under the supervision of the lead researcher, discussed and reviewed their versions to create a synthesized translation. This process resulted in a single preliminary version of the ICOAP-Knee in Brazilian Portuguese.

Stage 3: The synthesized Brazilian Portuguese version of the ICOAP-Knee was then subjected to back-translation. Two additional independent translators (Translator 3 and Translator 4), both fluent in Portuguese and English and lacking prior knowledge of the health field, conducted the back-translation into English. Importantly, neither translator had access to the original English version of the questionnaire.

Stage 4: A panel composed of four physiotherapists, each with about ten years of experience in musculoskeletal rehabilitation, met with the four translators involved in the previous stages to review all translated and back-translated versions. Through a consensus process, and with the agreement of all participants, a version identified as the pre-final Brazilian Portuguese version of the ICOAP-Knee was established.

Stage 5: In accordance with the study’s inclusion criteria, the pre-final version of the ICOAP-Knee was administered to thirty participants. Each participant read and completed the questionnaire, and each item was evaluated as either “understandable” or “not understandable”. When an item was considered unclear, participants were asked to provide a reason. Items that were not understood by more than 20% of participants were reformulated and subsequently retested with a new sample of thirty individuals [5].

### 2.5. Phase 3

This phase assessed the validity of the final version of the ICOAP adapted for cross-cultural use in Brazilian Portuguese. With the finalized Brazilian Portuguese version of the ICOAP-Knee, Phase 3 involved evaluating its measurement properties, including structural validity, convergent validity, internal consistency, floor and ceiling effects, and test–retest reliability over a 7-day interval [8]. A total of 103 individuals were included in the analyses of structural validity, convergent validity, internal consistency, and floor and ceiling effects, while a subsample of 53 participants was used to assess test–retest reliability.

Subsequently, the ICOAP-Knee was used alongside other PROMs, including the Numeric Rating Pain Scale (NRPS), the International Knee Documentation Committee (IKDC) questionnaire, and the Short Form-36 Health Survey (SF-36). These PROMs were selected for their clinical relevance, rigorous validation, and robust psychometric properties, ensuring a comprehensive assessment of pain intensity, physical function, and quality of life in individuals with KOA.

The NRPS is adaptable to various cultures and languages. It consists of a numerical scale from 0 to 10, where 0 indicates “no pain” and 10 represents “the worst pain imaginable”. Pain intensity is assessed based on the individual’s experience over the seven days prior to the evaluation [9].

The IKDC questionnaire is a tool designed to assess knee function and detect changes in symptoms, functionality, and sports activities resulting from knee impairments. It captures signs, symptoms, and disabilities associated with conditions such as anterior cruciate ligament injuries, meniscal lesions, and osteoarthritis. The instrument has been translated and culturally adapted for Brazilian Portuguese [4]. It consists of 18 items distributed across three domains: (1) symptoms, which includes seven items related to pain, swelling, stiffness, and weakness; (2) sports and daily activities, covering one item on sports participation and nine items on daily activities; and (3) current knee function and pre-injury function, which comprises one item (not included in the total score). Response formats vary across items: item 6 uses a yes/no response; items 1, 4, 5, 7, 8, and 9 utilize a 5-point Likert scale; and items 2, 3, and 10 employ an 11-point numeric scale. The final score is calculated on a scale from 0 to 100 using the total sum of valid item scores divided by the number of valid items. Higher scores indicate better knee function [10].

The Short Form-36 Health Survey (SF-36) is a generic instrument for assessing quality of life. It comprises eight domains: physical functioning, role limitations due to physical health, bodily pain, general health perceptions, vitality, social functioning, role limitations due to emotional problems, and mental health. Each domain is scored on a scale from 0 to 100, with higher scores indicating better health status. The SF-36 has been culturally adapted and validated for use in Brazilian Portuguese [11].

After confirming the diagnosis, a physiotherapist was responsible for recruiting and assigning participants, while a second researcher administered the questionnaires. Data analysis was conducted independently by a third researcher. The questionnaires were administered individually, without a time limit, either through face-to-face interviews or video calls. All researchers involved had an average of 10 years of experience in physiotherapy and specialized expertise in managing chronic musculoskeletal pain. Additionally, they underwent three months of training prior to the study to ensure consistency and accuracy in the assessments.

### 2.6. Statistical Analysis

The Kolmogorov–Smirnov test was utilized to verify the data distribution and was supported by the analysis of the related histogram plots. Due to the observed non-normality of the data distribution in the sample examined, we employed the Spearman correlation coefficient (rho). Sociodemographic data and PROM scores were summarized using the median and standard deviation (SD).

An exploratory factor analysis (EFA) was conducted using polychoric correlations and a robust diagonally weighted least squares (RDWLS) extraction method based on parallel analysis. This method is more accurate than other commonly used techniques, such as those that rely on an eigenvalue greater than one and the inflection point of the scree plot. Parallel analysis assesses the explained variance of the random correlation matrices generated by the software in comparison to the variance of the actual data matrix. The variance of each observed variable is evaluated against its associated random variance. Factors related to observed variance that exceed the random variance should be retained [12,13].

The following exploratory factor analysis (EFA) was performed using confirmatory factor analysis (CFA). Additionally, the analysis employed a polychoric covariance matrix and the robust diagonally weighted least squares (RDWLS) extraction method. The following indices were evaluated to assess model fit: comparative fit index (CFI), Tucker–Lewis index (TLI), root mean square error of approximation (RMSEA), standardized root mean square residual (SRMR), and chi-square/degrees of freedom. To determine model acceptability, the criteria were established as CFI and TLI > 0.90, RMSEA and SRMR < 0.08, and chi-square/GL < 3 [14,15].

Internal consistency was evaluated using Cronbach’s alpha and the alpha value if an item was omitted. Alpha values equal to or greater than 0.70 but less than 0.95 were considered adequate [16].

Test–retest reliability was analyzed using the intraclass correlation coefficient (ICC) and the 95% confidence interval (CI). Reliability was determined using ICC type 2,1 along with 95% CIs. ICC values lower than 0.40 indicate poor reliability; those between 0.40 and 0.75 indicate moderate reliability; values between 0.75 and 0.90 indicate substantial reliability; and those greater than 0.90 indicate excellent reliability [17]. Two measures were employed to assess agreement: SEM, standard error of measurement, and MDC, minimum detectable change. The following formula was used to calculate the SEM: SD × √ (1 − ICC). We applied the formula 1.96 × SEM × √2 to calculate the MDC [18]. The percentage of SEM relative to the total scale score was interpreted as very good when ≤5%, good when >5% and ≤10%, doubtful when >10% and ≤20%, and poor when >20%. Values exceeding the MDC indicate a change in a patient’s score that surpasses measurement error [14,16].

Convergent validity was assessed using the Spearman correlation coefficient (rho) test, where r < 0.30 indicated a weak correlation, r ≥ 0.30 and <0.60 indicated a moderate correlation, and r ≥ 0.60 indicated a good correlation [19].

The ceiling and floor effects of the ICOAP hip were also examined. These effects occur when more than 15% of participants achieve the minimum or maximum score [14,16].

We tested the hypothesis that the strength of the correlation with the Brazilian Portuguese version of the ICOAP was ≥0.60 for its relationship with the NPRS and IKDC. For the SF-36 domains, this correlation ranged from ≥0.30 to <0.60. Although inverse correlations were found between the ICOAP, IKDC, and SF-36, correlations in the same direction were observed with the NPRS.

Software Factor (version 10.10.03, Universitat Rovira i Virgili, Tarragona, Catalonia, Spain) was used for exploratory factor analysis (EFA), while RStudio (version 1.1.453, Boston, MA, USA), along with the packages Lavaan (version 0.65) and SemPlot (version 1.1.2), was employed for confirmatory factor analysis (CFA). Additionally, SPSS (version 20, Chicago, IL, USA) was utilized to analyze the remaining measurement properties and conduct descriptive analysis.

## 3. Results

The pre-final version of the ICOAP was administered to 30 individuals with KOA who speak Brazilian Portuguese as their first language. None of the ICOAP items were misunderstood by more than 20% of the participants. Therefore, the final version of the ICOAP in Brazilian Portuguese was created, closely following the original version.

A total of 103 individuals diagnosed with KOA participated in the study (Table 1). Table 1 provides a descriptive analysis of the questionnaires utilized in the study. Most participants were female, married, had not completed high school, were engaged in a professional activity, had the left limb affected, and had been diagnosed over 10 years ago. The majority reported experiencing constant pain as the most common symptom and were receiving physiotherapy treatment.

Table 2 presents the results for convergent validity, indicating that the ICOAP constant pain domain correlated with all questionnaires used in the study, with classifications ranging from moderate to good. In contrast to the constant pain domain, the intermittent pain domain exhibited statistically significant correlations only with the IKDC questionnaire and the social aspects domain. However, these correlations fell below the classification threshold referenced in this study. The internal consistency values for both domains were considered adequate at 0.98 (Table 2).

Also, in Table 2, the test–retest reliability (ICC_2,1_) was rated as excellent for both ICOAP domains, with values of 0.95 for the constant pain domain and 0.92 for the intermittent pain domain. Regarding measurement error, in addition to high MDC and SEM values, the SEM (%) values were particularly noteworthy, being classified as doubtful for the constant pain domain (15.99) and poor for the intermittent pain domain (41.00).

**Figure 1:**
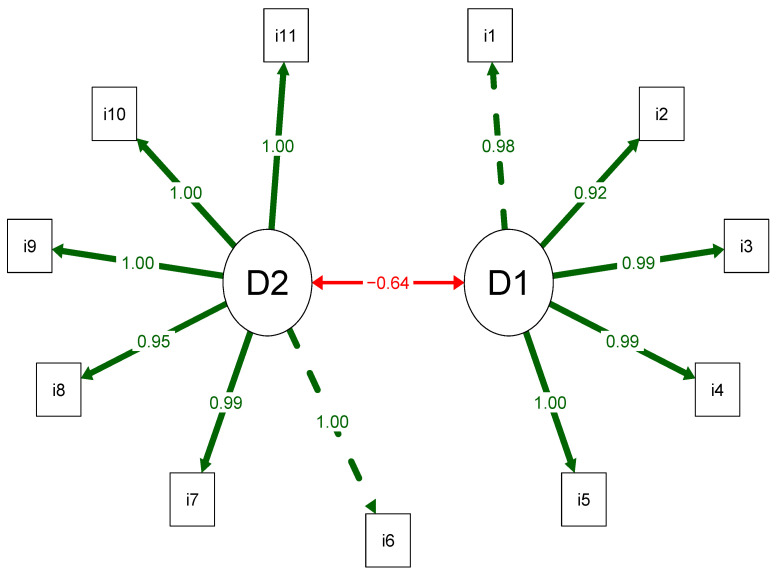
Path diagram of the Brazilian Portuguese IC, with two domains and 11 items.

The structural analysis results indicate that the original ICOAP structure, which comprises two domains and 11 items, shows adequate fit for all indices: chi-square/GL = 1.48; CFI = 1.00; TLI = 1.00; RMSEA (90% CI) = 0.068 (0.027 to 0.102); and SRMR = 0.045. Furthermore, the factor loadings demonstrating the relationship between the domain and the items were higher than the acceptability cutoff point (>0.40) (Figure 1).

## 4. Discussion

The present study aimed to translate, culturally adapt, and validate the ICOAP for use in Brazilian Portuguese among individuals with knee osteoarthritis (KOA). The final version of the instrument was reported to be easy to understand, with no item exceeding the 20% incomprehension threshold among participants. These findings suggest that the Brazilian Portuguese version of the ICOAP may serve as a valuable addition to the available PROMs for assessing pain in the Brazilian KOA population.

The ICOAP has been translated and validated in multiple languages, including English, Traditional and International Chinese, Turkish, Arabic, Persian, German, Czech, Dutch, French, Italian, Norwegian, European Portuguese, Spanish, North and Central American Spanish, and Swedish [20,21,22,23,24,25,26,27]. Compared to other PROMs, the ICOAP provides a more comprehensive approach to evaluating pain in individuals with KOA. Its primary strength lies in its ability to capture the multidimensional nature of chronic pain by distinguishing between constant and intermittent pain and addressing its functional and behavioral consequences [22,26].

Regarding previous versions of the ICOAP, there remains a need for greater standardization of comparator instruments to support assessments of convergent validity. To date, only the Turkish and Chinese versions have examined correlations between the ICOAP domains and pain intensity [21,22]. Furthermore, this is the first study to explore the relationship between the constant and intermittent pain domains of the ICOAP and quality-of-life constructs. It is important to note that self-reporting instruments often lack a universally accepted gold standard. Therefore, convergent validity must be assessed using measures of the same construct, a related but different construct, or even a distinct construct that is theoretically expected to correlate with the target domain [25].

Based on this context, the predefined hypotheses were partially confirmed. The convergent validity analysis revealed significant correlations between the ICOAP constant pain domain and all the assessment instruments used. In contrast, the intermittent pain domain showed weaker and less consistent correlations. We speculate that these findings may be explained by the fact that constant pain tends to have a more profound and lasting impact, making it more memorable and continuously influential on functionality, quality of life, and overall well-being in individuals with KOA. Conversely, due to its fluctuating nature, intermittent pain may exert more specific and less intense effects on these outcomes. This is likely because individuals with KOA may develop greater coping strategies and self-management skills when dealing with intermittent pain episodes [7,28].

One of the key features of the ICOAP is its ability to capture the impact of chronic pain, characterized as constant and/or intermittent, on functional outcomes and overall well-being. The relevance of distinguishing these domains becomes increasingly evident, particularly when considering the results of the structural validity analysis. To our knowledge, this is the first study to conduct such an analysis of the ICOAP-Knee. The two-dimensional structure of the Brazilian Portuguese version of the ICOAP was confirmed, with all 11 items demonstrating adequate fit indices, indicating that the instrument accurately reflects the dimensionality of the construct [21]. This supports the potential for a multifaceted analysis of pain perception, not only by accounting for the intensity and consequences of chronic pain but also by recognizing its dynamic nature, as it may present either as constant or intermittent depending on the context.

Additionally, high values were observed for ICC (2,1) and Cronbach’s alpha in both the constant and intermittent pain domains, confirming that the Brazilian Portuguese version of the ICOAP has a satisfactory internal structure and reliable application. These results are consistent with findings from the original version [2], as well as the Turkish [21], Portuguese [26], Chinese [27], and Greek [29] adaptations. Test–retest reliability is established by administering the same instrument to the same individuals twice within a predetermined time interval [30]. In the context of ICOAP validation studies, this time interval has varied. Most studies opt for a reapplication period of 2 to 5 days [26,27]. When longer intervals were used—such as two weeks—the reliability coefficients, particularly for the intermittent pain domain, tend to be lower compared to those in studies with shorter intervals [30,31].

It is important to note that although the ICOAP has already been translated into European Portuguese [26], this version may not be fully applicable to the Brazilian context. Despite sharing the same root language, Brazilian Portuguese and European Portuguese differ substantially in vocabulary, expressions, and linguistic structures, which may affect comprehension and response accuracy in self-reported questionnaires. Furthermore, Brazil has distinct sociocultural and healthcare system characteristics, including differences in access to healthcare services, average education levels, health literacy, treatment adherence, and culturally specific expressions of pain [32,33]. These factors highlight the need for a culturally and linguistically adapted version of the ICOAP specifically tailored to the Brazilian population to ensure content validity and the appropriate interpretation of each item [32,33].

Although it was expected that the ICOAP intermittent pain domain would show strong correlations with all SF-36 domains, particularly those related to physical functioning, vitality, and pain, the observed correlations were weak or non-significant. These findings do not directly indicate poor construct validity but may instead reflect the multidimensional and episodic nature of intermittent pain, which is less consistently captured by generic health-related quality-of-life instruments like the SF-36. Furthermore, we believe that episodes of intermittent pain may have a more variable and transient impact, making their effects less directly aligned with the broader and more stable constructs assessed by the SF-36 [34]. Therefore, although the weak correlations suggest a limited degree of convergent validity with the selected comparator measures, they should not be interpreted as definitive evidence of inadequacy. Rather, these results highlight the need for complementary assessments and may point to the specificity of the intermittent pain construct, which may require more tailored instruments or domain-specific comparators for more accurate validation [34].

The values associated with measurement error were deemed inadequate, raising concerns about the precision of the instrument and its ability to detect true changes within the sample. These variations may be attributed to difficulties in recognizing and differentiating between constant and intermittent pain. This challenge is likely related to the fact that most participants in this study were undergoing rehabilitation, which may have contributed to fluctuations in their pain levels and perceptions between assessment points.

The high SEM and MDC values suggest considerable variability in measuring the constructs. These results may reflect the difficulty in understanding the manifestations of constant and intermittent pain. However, they also raise important considerations for both research and clinical use of the assessment instrument. Such values indicate that relatively large changes in scores are needed to surpass the threshold of measurement error and be interpreted as a true change in the patient’s condition. This limits the instrument’s sensitivity in detecting small but potentially meaningful changes. Therefore, the use of the instrument may be more appropriate in longitudinal studies with longer follow-up periods or in clinical trials evaluating interventions expected to produce direct and substantial effects [35].

This study presents several limitations that should be acknowledged. First, the use of convenience sampling may limit the generalizability of the findings since the sample may not adequately represent the broader population of individuals with KOA. The participants were exclusively those undergoing physiotherapeutic treatment, which may have introduced selection bias by excluding individuals who are not actively engaged in rehabilitation services or who receive care through other modalities. This could potentially affect the external validity of the results. Second, certain psychometric analyses were not performed, highlighting important areas for future investigation. In particular, the study did not assess inter-rater reproducibility, which would have provided valuable insights into the consistency of the instrument used by different evaluators. The absence of this analysis limits conclusions regarding the general reliability of the tool across varied clinical settings and raters. Furthermore, although classical test theory methods were used to assess measurement properties, applying modern psychometric techniques, such as Rasch analysis, could offer more refined information regarding item functioning, measurement invariance, and scale dimensionality. Future research should consider incorporating such methodologies to strengthen the robustness and precision of validation efforts.

## 5. Conclusions

The Brazilian Portuguese version of the ICOAP-Knee includes two independent domains, both of which demonstrated adequate internal consistency and reliability. However, only the constant pain domain showed acceptable levels of convergent validity with instruments assessing pain intensity, function, and quality of life. Both domains exhibited measurement error values that exceeded commonly accepted thresholds.

## Figures and Tables

**Table 1:** Characteristics of the study participants (n = 103).

Variables	Number, Median (SD)
Age (years)	60.64 (10.04)
Body mass (Kg)	78.33 (15.54)
Stature (m)	1.64 (6.23)
BMI (Kg/m^2^)	28.78 (6.34)
Sex	
Male	7
Female	96
Marital status	
Single	12
Married	53
Widower	17
Divorced	21
Education	
University incomplete	11
University complete	15
High school incomplete	38
High school complete	15
Elementary school	24
Professional activity	
Active	67
Inactive	36
Affected member	
Right	39
Left	43
Bilateral	21
Diagnosis time (years)	11.02
Type of pain reported	
Intermittent	18
Constant	62
Both	23
Physiotherapeutic treatment	
Yes	82
No	21
NRPS (0–10 points)	6.16 (2.53)
IKDC (0–100 points)	33.69 (16.82)
ICOAP	
Constant (0–100 points)	44.51 (30.42)
Intermittent (0–100 points)	14.64 (21.17)
SF 36	
Functional capacity (0–100 points)	33.49 (26.32)
Physical aspects (0–100 points)	33.98 (45.62)
Pain (0–100 points)	42.52 (21.09)
General health status (0–100 points)	43.39 (22.31)
Vitality (0–100 points)	42.91 (13.69)
Social aspects (0–100 points)	53.76 (22.87)
Emotional aspects (0–100 points)	56.95 (50.62)
Mental health (0–100 points)	51.37 (12.54)

BMI: body mass index, m: meters, Kg: Kilogram, SD: standard deviation, NPRS: Numeric Pain Rating Scale (high score = higher level of intensity of pain), IKDC: International Knee Documentation Committee (high score = higher level of knee function), ICOAP: intermittent and constant osteoarthritis pain (high score = higher level of constant and intermittent of pain), SF-36: 36-Item Short Form Survey (high score = higher level of quality of life).

**Table 2:** Convergent validity and internal consistency of ICOAP constant and intermittent pain domains.

Properties	Values		Classification	
	ICOAP—Constant Pain	ICOAP—Intermittent Pain	ICOAP—Constant Pain	ICOAP—Intermittent Pain
Convergent validity (n = 103)				
NRPS (0–10 points)	0.70 **	−0.15	Good	-
IKDC (0–100 points)	−0.77 **	0.19 *	Good	-
SF-36				
Functional capacity (0–100 points)	−0.67 **	0.18	Good	-
Physical aspects (0–100 points)	−0.47 **	−0.20	Moderate	-
Pain (0–100 points)	−0.77 **	0.20 *	Good	-
General health status (0–100 points)	−0.58 **	0.10	Moderate	-
Vitality (0–100 points)	−0.39 **	0.64	Moderate	-
Social aspects (0–100 points)	−0.69 **	−0.26 **	Good	-
Emotional aspects (0–100 points)	−0.27 **	0.41	-	-
Mental health (0–100 points)	−0.49 **	0.16	Moderate	-
Internal consistency (n = 103)				
Cronbach’s alpha	0.98	0.98	Adequate	Adequate
Test–retest reliability (n = 53)				
Median (SD)—1	46.94 (31.05)	15.74 (23.36)		
Median (SD)—2	41.94 (29.55)	14.12 (19.92)		
ICC_2,1_	0.95	0.92	Excellent	Excellent
SEM (points)	7.11	6.12		
SEM (%)	15.99	41.00	Doubtful	Poor
MDC (points)	19.70	16.97	-	-
MDC (%)	44.32	113.64	-	-

** Significant correlation (*p* < 0.01). * Significant correlation (*p* < 0.05). NPRS: Numeric Pain Rating Scale (high score = higher level of intensity of pain); IKDC: International Knee Documentation Committee (high score = higher level of knee function); ICOAP: intermittent and constant osteoarthritis pain (high score = higher level of constant and Intermittent of pain); SF-36: 36-Item Short Form Survey (high score = higher level of quality of life); SD: standard deviation; ICC2, 1: intraclass correlation coefficient 2, 1; SEM: standard error of measurement; MDC: minimal detectable change.

## Data Availability

The data that support the findings of this study are available from the corresponding author (PF) upon reasonable request.
